# Optogenetic Stimulation of G_i_ Signaling Enables Instantaneous Modulation of Cardiomyocyte Pacemaking

**DOI:** 10.3389/fphys.2021.768495

**Published:** 2021-12-20

**Authors:** Milan Cokić, Tobias Bruegmann, Philipp Sasse, Daniela Malan

**Affiliations:** ^1^Medical Faculty, Institute of Physiology I, University of Bonn, Bonn, Germany; ^2^Research Training Group 1873, University of Bonn, Bonn, Germany

**Keywords:** optogenetics, cardiomyocyte, GPCR (G protein coupled receptor), G_i_ signaling pathway, GIRK channel, pacemaking

## Abstract

G-protein signaling pathways are central in the regulation of cardiac function in physiological and pathophysiological conditions. Their functional analysis through optogenetic techniques with selective expression of opsin proteins and activation by specific wavelengths allows high spatial and temporal precision. Here, we present the application of long wavelength-sensitive cone opsin (LWO) in cardiomyocytes for activation of the G_i_ signaling pathway by red light. Murine embryonic stem (ES) cells expressing LWO were generated and differentiated into beating cardiomyocytes in embryoid bodies (EBs). Illumination with red light (625 nm) led to an instantaneous decrease up to complete inhibition (84–99% effectivity) of spontaneous beating, but had no effect on control EBs. By using increasing light intensities with 10 s pulses, we determined a half maximal effective light intensity of 2.4 μW/mm^2^ and a maximum effect at 100 μW/mm^2^. Pre-incubation of LWO EBs with pertussis toxin completely inhibited the light effect proving the specificity for G_i_ signaling. Frequency reduction was mainly due to the activation of GIRK channels because the specific channel blocker tertiapin reduced the light effect by ~80%. Compared with pharmacological stimulation of M_2_ receptors with carbachol with slow kinetics (>30 s), illumination of LWO had an identical efficacy, but much faster kinetics (<1 s) in the activation and deactivation demonstrating the temporal advantage of optogenetic stimulation. Thus, LWO is an effective optogenetic tool for selective stimulation of the G_i_ signaling cascade in cardiomyocytes with red light, providing high temporal precision.

## Introduction

G-Protein-coupled receptors (GPCR) play a pivotal role in regulating cardiac function. The counteracting effects of G_s_ and G_i_ proteins are fundamental for the heart rate regulation, contractility, neurohormonal control of circulatory system, and in pathophysiological conditions (Capote et al., 2015). As an antagonist of G_s_-pathway, the G_i_-pathway inhibits the adenylate cyclase, decreasing intracellular cAMP level, diminishing protein kinase A (PKA) activity, and thus, reducing L-type Ca^2+^ currents. In atrial myocytes and cells of the sinus node and AV node, acetylcholine opens a type of inwardly rectifying potassium channel (I_K,ACh_/GIRK) through direct effects of the G_i_ βγ subunit (Krapivinsky et al., 1995; Ivanova-Nikolova et al., 1998). This results in hyperpolarization, slowing of heart rate, prolongation of AV-node conduction, and shortening of the action potential duration in atrial cardiomyocytes (Belardinelli and Isenberg, 1983). Some G_i_ coupled receptors, such as adenosine-A1, are cardioprotective (Hutchinson and Scammells, 2004) and reduce the risk of cardiac arrhythmia, moreover G_i_-coupled α_2_ receptors exert an important sympatho-inhibitory function (Xiang and Kobilka, 2003).

Optogenetics is a photostimulation technique which selectively activates opsin proteins by specific wavelengths and the use of light has a much higher spatial and temporal precision than application and diffusion of receptor agonists (Beiert et al., 2014; Guru et al., 2015; Makowka et al., 2019). For instance, the blue light-sensitive receptor Jellyfish Opsin has been used for selective activation of G_s_ signaling cascade in cardiomyocytes and the heart with high spatial and temporal precision (Makowka et al., 2019), but direct control of the G_i/o_ signaling cascade in cardiomyocytes with light has not been shown yet.

Short- and long-wavelength-sensitive opsins (LWO) have been used before for control of neuronal G_i/o_ signaling pathways (Masseck et al., 2014) and LWO has been shown to activate rather G_i/t_ than G_o_ signaling (Ballister et al., 2018). Thus, the aim of this study was to use the red light-activated LWO to stimulate G_i_ signaling pathways in cardiomyocytes.

## Methods

### Vector Construction and Transfection of Embryonic Stem (ES) Cells

An LWO-enhanced yellow fluorescent protein (eYFP) plasmid was generated for the expression of LWO (human long-wavelength-sensitive opsin 1, NP_064445.1) c-terminally fused with eYFP using codon optimized synthesized DNA (GeneArt, Life Technologies, Germany) excised with SpeI und MluI and subcloned into a backbone vector with the chicken β-actin promoter (CAG) described before (Beiert et al., 2014). In this study, 40 μg of DNA was linearized with Bgl II and electroporated into 4 × 10^6^ mouse embryonic stem (ES) cells (D3 line) with a single electrical pulse (250 V, 750 μF, 0.4 cm electrode gap, BioRad Gene Pulser, CA, USA). The electroporated cells were plated and 400 μg/ml neomycin was added for selection 24 h after transfection. The eYFP positive clones were picked and cultured separately. Two positive clones were chosen, because of their specific eYFP expression in cardiomyocytes and stable light reaction. As a control group, we used the D3 ES cells with stable expression of the enhanced green fluorescent protein (eGFP) under the control of the CAG promoter as reported before (Beiert et al., 2014).

### ES Cell Culture and Differentiation

Embryonic stem cells were cultured and differentiated within embryoid bodies (EBs) using the hanging drop method as previously described (Bruegmann et al., 2010; Beiert et al., 2014). For differentiation, we used Iscove's Modified Eagle's Medium (Invitrogen, MA, USA) supplemented with 20% fetal calf serrum (FCS) (Pan-Biotech, Germany), 0.1 mM non-essential amino acids (Invitrogen), 100 U/ml penicillin (Invitrogen), 100 mg/ml streptomycin (Invitrogen), and 0.1 mmol/L β-mercaptoethanol (Sigma Aldrich, MO, USA). At day 5 of differentiation, EBs were either plated on 0.1% gelatin-coated glass cover slips for the analysis of beating frequency conducted at day 9–14 or fixated for immunohistochemical analysis. In some differentiations, the method was modified by adding Noggin (R&D System, MN, USA, 250 ng/ml) from day 4 to 6 and retinoic acid (Sigma Aldrich, 1 μM) from day 6 to 8 and Dickkopf-related protein 1 (R&D system, 200 ng/ml) from day 6 to 11.

### Immunofluorescence

Embryoid bodies were fixed with 4% paraformaldehyde (Sigma Adrich) and permeabilized with 0.2% of Triton X-100 (Sigma Adrich) for 20 min, blocked with 5% of donkey serum (Jackson ImmunoResearch, UK) for 20 min and stained for 2 h at room temperature with primary antibody against α-actinin (1:400, Sigma Aldrich). Alexa Fluor 647 conjugated secondary antibody (1:400, Invitrogen) diluted in 1 μg/ml Hoechst 33342 (Sigma Aldrich) was applied for 1 h at room temperature. The pictures were taken with an Eclipse Ti2 microscope with NIS-Elements and deconvolution software (Nikon, Japan).

### Frequency Analysis and Light Stimulation of EBs

Beating EBs that showed eYFP expression in beating areas were used at day 9–14 of differentiation. Then, 1–2 h before experiment, the medium was replaced with Tyrode external solution (in mM: 142 NaCl, 5.4 KCl, 1.8 CaCl_2_, 2 MgCl_2_, 10 glucose, and 10 HEPES; pH 7.4) containing 11-cis retinal (1 μM, Santa Cruz Biotechnology, TX, USA) or 9-cis retinal (1 μM, Sigma Aldrich). Video microscopy of beating EBs was performed while perfusing EBs with Tyrode solution without retinal at ~35°C on an Axiovert 200 microscope with a 5× objective (Fluar, NA 0.25, Zeiss, Germany) using infrared light (760 nm, 1.8 μW/mm^2^ at the focal plane) to avoid accidental LWO activation. Spontaneous contraction was recorded with a charge-coupled device (CCD) camera (piA640-210gm, Basler, Germany) at 51 fps and analyzed online using custom designed software (LabView, National Instruments, TX, USA) as described before (Bruegmann et al., 2010; Makowka et al., 2019). Optogenetic stimulation was performed with a 625 nm LED within the LedHUB (Omicron Laserage, Germany) equipped with a 10% neutral density filter and coupled to the objective with an optical fiber and a 660 nm dichroic filter (AHF Analysentechnik, Germany). Illumination was controlled by a recording system (PowerLab 4/35 and Labchart software, AD Instruments, Sydney, Australia), which was also used to record time points of individual beats to calculate the frequency. Light intensity was calibrated at the objective with a power meter (PM100 power meter, S130A sensor, Thorlabs, NJ, USA) before each experiment.

### Analysis of LWO Effect and Light Sensitivity

For statistical analysis, only stable beating EBs without arrhythmical episodes over 5 min before illumination were included. The average frequency during 10 s continuous illumination was analyzed and normalized to baseline 10 s before. To describe the effectivity of LWO, we calculated the blocking effect with 100% as complete block and 0% as no effect on beating frequency. To determine light sensitivity, the light intensity of individual light pulses was gradually increased (in μW/mm^2^: 0.3; 1; 3; 10; 30; 100; and 300). In addition, 100 μW/mm^2^ individual light pulses of increasing durations (0.1; 0.3; 0.5; 1; 3; 5; 10; and 30 s) were applied and the frequency was averaged 5 s after onset of illumination compared with 5 s before. Half maximal effects were analyzed by fitting the normalized frequency with a Hill function with upper level set to 100% (GraphPad Prism, CA, USA).

### Pharmacology

To compare LWO effect with pharmacological activation, we used supramaximal illumination (100 μW/mm^2^, 10 s) and stimulation of the muscarinergic acetylcholine receptor with carbachol (60 s, 100 μM, Sigma Aldrich). Effectiveness of the inhibition was calculated 5 s after the lowest frequency (to account for variations in perfusion kinetics) compared with 5 s before illumination/perfusion. The delay to maximum block was calculated between onset of illumination/perfusion and maximal effect and the delay to 50% recovery as end of stimulation/perfusion until frequency reached 50% of initial baseline. To block G_i_ proteins, pertussis-toxin (PTX, Invitrogen) was applied for 24 h in IMDM medium without FCS at a final concentration of 0.5 μg/ml. The blocker of G protein-coupled inwardly-rectifying potassium channels (GIRK) tertiapin-q (100 nM, Tocris, Bristol, UK) was incubated for 30 min after one initial measurement and kept in the solution for the second measurement (**Figure 2C**).

### Statistics

**Figure 1:**
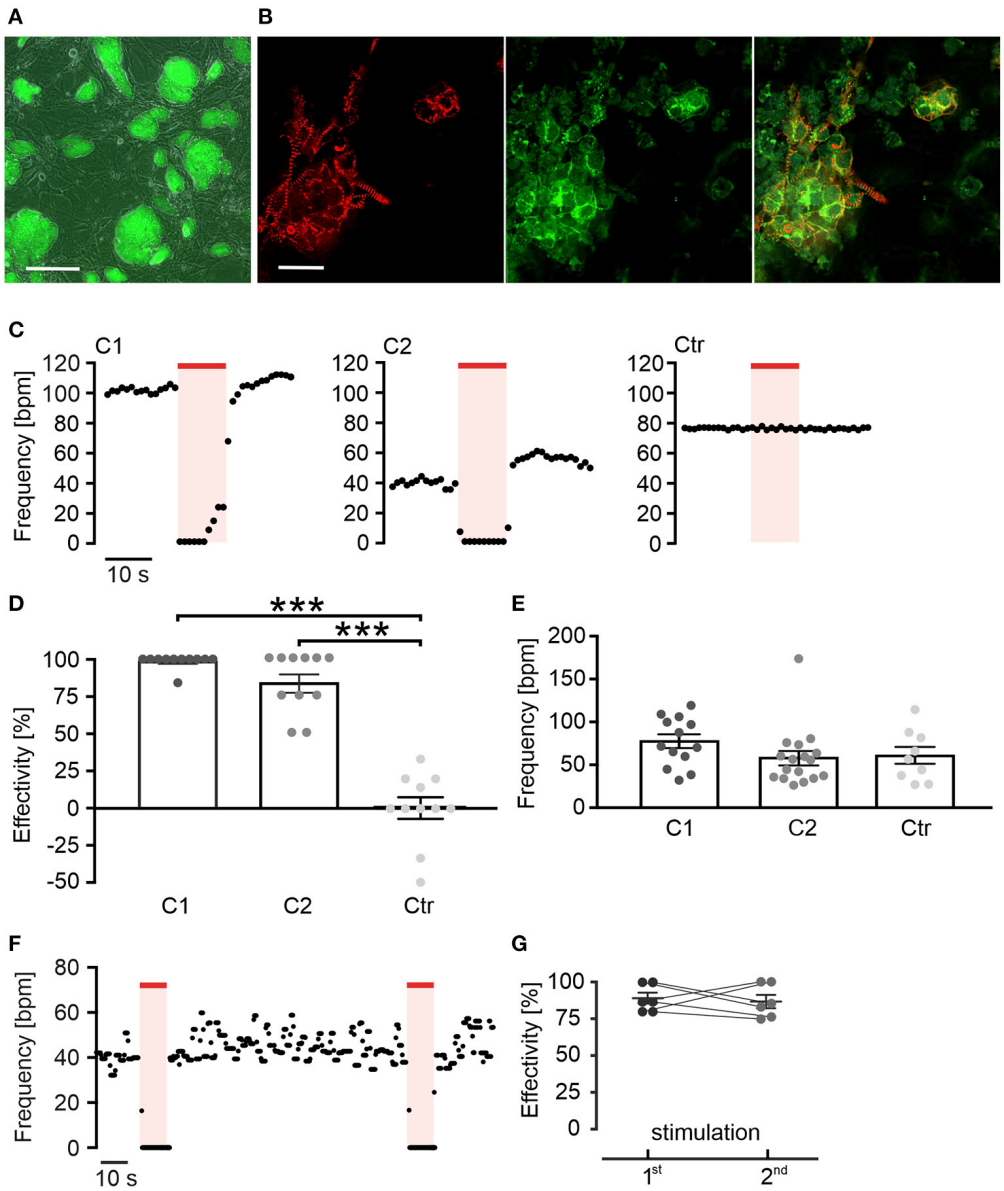
Red light activation of long wavelength-sensitive cone opsin (LWO) decreases beating frequency of cardiomyocytes. **(A)** Embryonic stem (ES) cell colonies with LWO expression indicated by eYFP fluorescence (green, bar = 200 μm). **(B)** EYFP fluorescence (green) in α-actinin (red) positive cardiomyocytes differentiated within an embryoid body (EB), generated from LWO ES cells (bar = 25 μm). **(C)** Representative frequency traces of spontaneous beating within two EBs differentiated from ES cell LWO clones (C1 and C2) and one EB from eGFP control ES cells (Ctr) stimulated with light (625 nm, 100 μW/mm^2^, red line). **(D)** Effectivity of inhibition (100% = complete block of spontaneous beating) by illumination [ANOVA Tukey's multiple comparison test: ***p < 0.001 (C1, C2, *n* = 11) vs. control (*n* = 11)]. **(E)** Statistical comparison of spontaneous beating frequency of EBs from two LWO clones and the wild-type ES cells [ANOVA Tukey's multiple comparison test, *p* = 0.98 (C2, *n* = 17) *p* = 0.46 (C1, *n* = 13) vs. control (*n* = 9)]. **(F)** Representative frequency trace of two supramaximal (10 s, 100 μW/mm^2^) repetitive light stimuli. **(G)** Comparison of frequency reduction effectivity of the first and the second light pulse (two side paired *t*-test: *p* = 0.73, *n* = 6).

**Figure 2:**
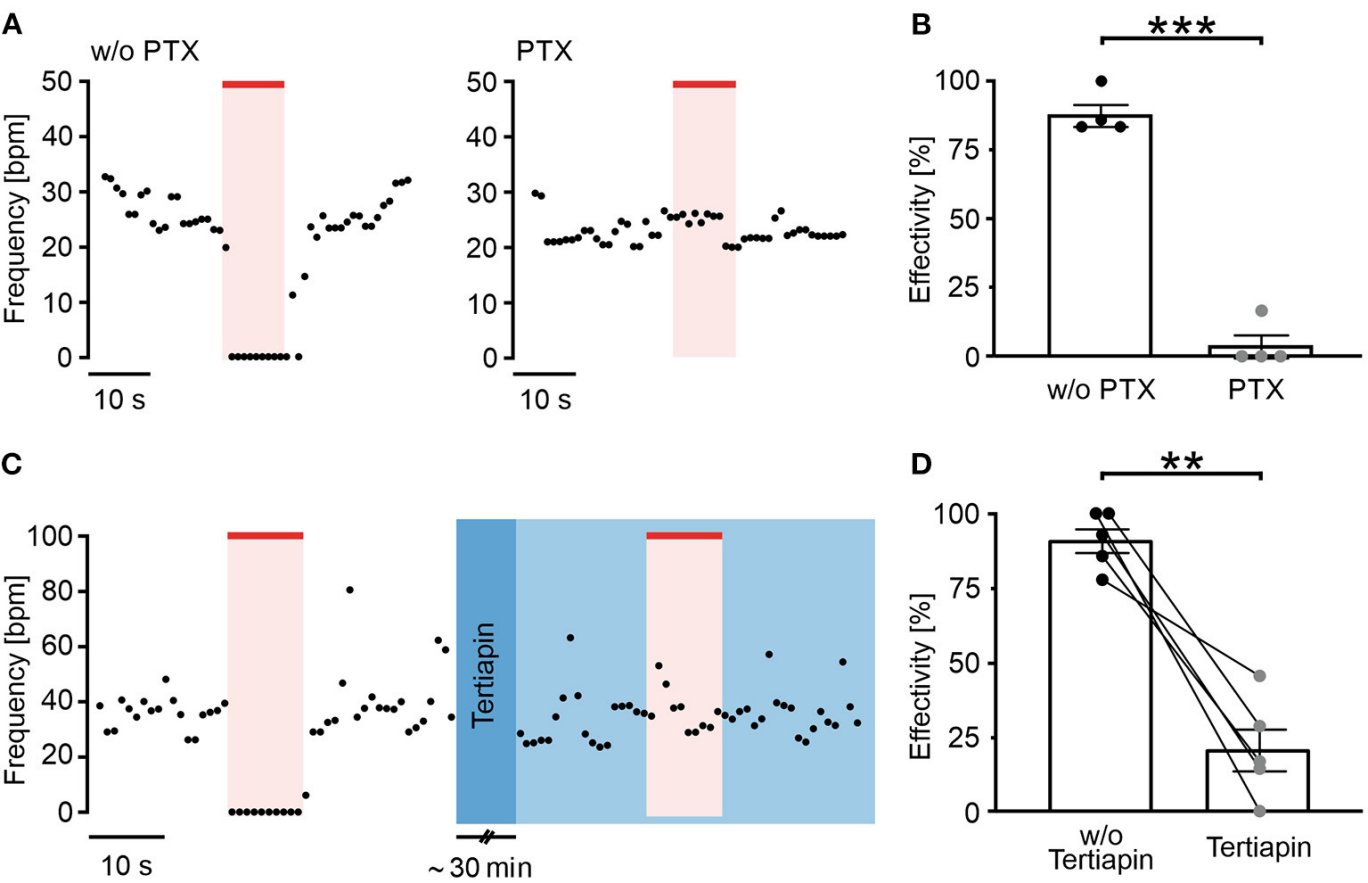
Long wavelength-sensitive cone opsin acts through G_i_ proteins and GIRK channels. **(A)** Representative frequency traces of LWO-EBs without (w/o, left) and with pertussis toxin (PTX) (0.5 μg/ml, 24 h, right) treatment with illumination (625 nm, 100 μW/mm^2^, red line). **(B)** Aggregated data of frequency reduction effectivity with and without PTX (unpaired *t*-test: ***p < 0.001, *n* = 4). **(C)** Representative frequency trace of an LWO-EB before (left) and after treatment with tertiapin (30 min incubation, right, blue) with illumination (625 nm, 100 μW/mm^2^, red line). **(D)** Statistical analysis of frequency reduction effectivity with or without tertiapin treatment (two side paired *t*-test: **p = 0.003, *n* = 5).

Data are shown as mean ± SEM and GraphPad Prism 7.0 was used to perform the statistical analysis. For frequency experiments in Figures 1D,E one-way ANOVA with Tukey's multiple comparison test was used. Pertussis toxin (PTX) effect (Figure 2B) was analyzed with an unpaired Student's *t*-test. For the other experiments (Figures 1G, 2D, **4B–D**), two-sided paired Student's *t*-tests were used. A *p*-value < 0.05 was considered statistically significant. The *n* values indicate the number of independent experiments (EBs).

## Results

### Red Light Activation of LWO Decreases Beating Frequency of Cardiomyocytes

The human LWO in fusion with eYFP was stably expressed under the control of the CAG promoter in ES-cells (D3 line) (Figure 1A). For differentiation of ES cells into spontaneously beating cardiomyocytes, EBs were generated with the hanging drop method (Wobus et al., 1991; Maltsev et al., 1993; Beiert et al., 2014). Spontaneously beating areas in the EBs showed differentiation into α-actinin and eYFP positive cardiomyocytes indicating LWO expression (Figure 1B). An analysis of beating frequency was performed by infra-red video microscopy to avoid LWO activation. Illumination of beating areas with red light pulses (λ = 625 nm, 10 s, 100 μW/mm^2^) led to an almost complete block of spontaneous beating in EBs from two separately differentiated ES cell clones (Figures 1C,D; clones C1 and C2). In contrast, illumination of EBs expressing only eGFP but not LWO did not reduce beating frequency (Figures 1C,D; Ctr). Importantly, the baseline beating frequency was similar between EBs from the two LWO clones and the control eGFP clone, suggesting that LWO expression does not negatively affect pacemaking and shows no dark activity (Figure 1E). Application of two identical light pulses with 90 s delay showed similar effectivity of beating block (Figures 1F,G) indicating that LWO can be activated repetitively without desensitization.

### LWO Activates G_i_-Proteins and GIRK Channels in Cardiomyocytes

To confirm the LWO specificity for G_i_ activation, EBs were pre-treated with PTX (0.5 μg/ml) for 24 h to block all G_i/o_ proteins. Subsequent frequency measurements showed that PTX almost completely inhibited the light effect (Figures 2A,B) and the blocking effectivity was only 4% in contrast to an effectivity of 88% in non-treated EBs recorded in parallel. Because activation of GIRK channels by G_i_-protein βγ subunits is the main mechanism for slowing the heart rate (Huang et al., 1995; Nobles et al., 2018), we compared light effects before and after application of the GIRK channel blocker tertiapin (100 nM, 30 min, Figure 2C, blue color) and found that tertiapin reduced the light effect significantly from 91 to 21% blocking effectivity (Figure 2D).

### Dose-Response Relationship Shows High Light Sensitivity of LWO

**Figure 3:**
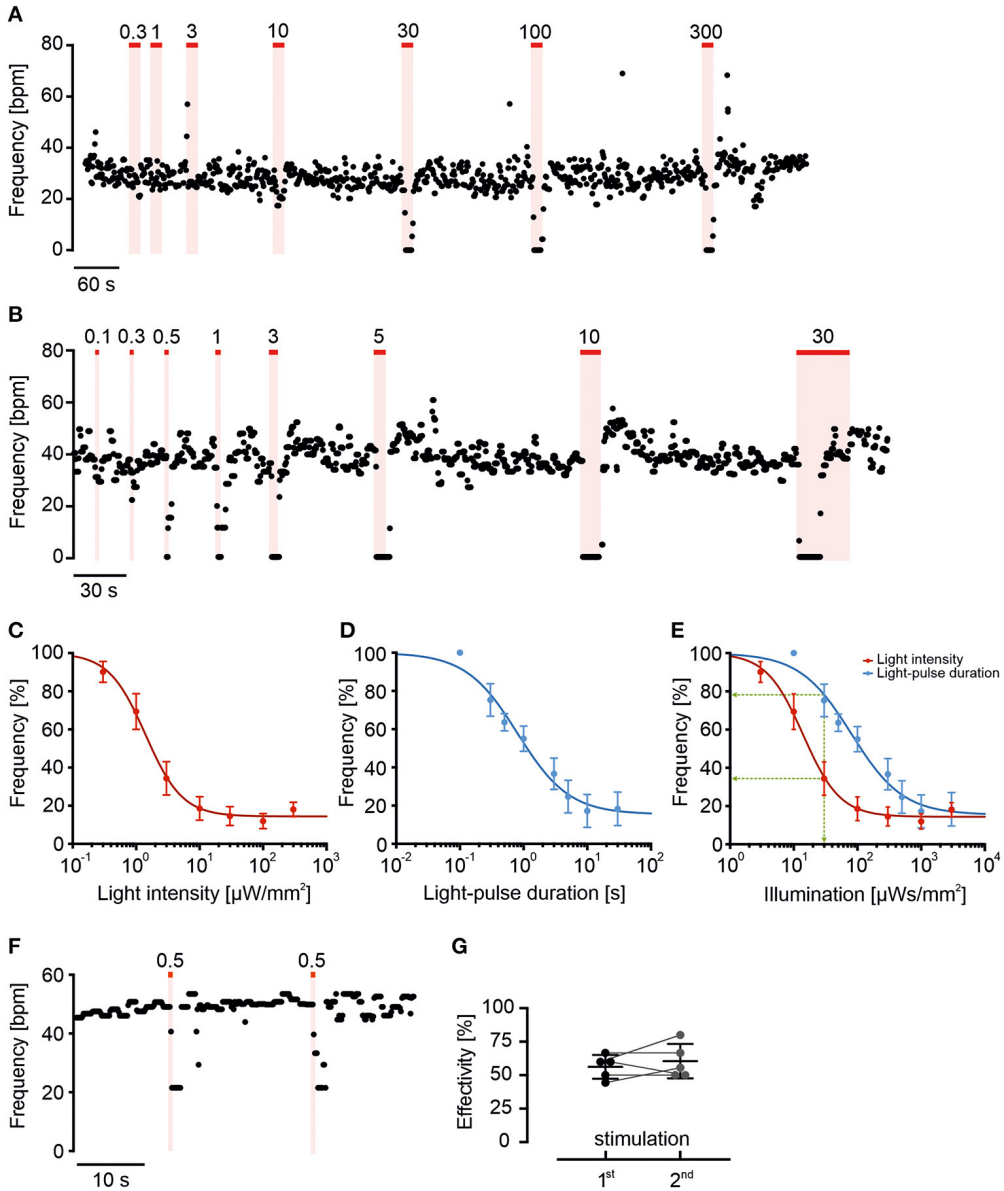
Long wavelength-sensitive cone opsin response can be fine-tuned by light intensity and duration. **(A,B)** Representative frequency traces of LWO-EBs with light stimulation (625 nm, red line) with increasing light intensities (**A**, 10 s light, indicated values in μW/mm^2^) or different durations [**(B)**, 100 μW/mm^2^, indicated pulse duration in s]. **(C,D)** Relationship between change in normalized beating frequency and light intensity [**(C)**, *n* = 9] or light pulse duration [**(D)**, *n* = 6] on a logarithmic scale fitted with Hill equation. **(E)** Relationship between change in beating frequency and total light energy calculated as s*μW/mm^2^ from data in **(C)** (red) and **(D)** (blue). Note that the effect of 30 s*μW/mm^2^ light energy (green line) is stronger using 10 s at 3 μW/mm^2^ (red) compared with 0.3 s at 100 μW/mm^2^ (blue). **(F)** Representative frequency trace and **(G)** statistical analysis of two submaximal (0.5 s, 100 μW/mm^2^) repetitive light stimuli (two side paired *t*-test: *p* = 0.46, *n* = 5).

To determine LWO light sensitivity, we repetitively applied light with increasing light energy by using ascending light intensities or durations (Figure 3). Application of 10 s long pulses with stepwise increasing light intensities from 0.3 to 300 μW/mm^2^ led to a gradual decrease of beating frequency after each light pulse (Figure 3A) with a sigmoidal dependency of the blocking effect on the logarithm of intensity (averaged data in Figure 3C). The data points of each individual experiment were fitted with the Hill equation resulting in an average half maximal effective light intensity (ELi50) of 2.4 ± 0.7 μW/mm^2^ (*n* = 9) and a maximum blocking effect at ~ 100 μW/mm^2^. Similarly, application of light pulses of 100 μW/mm^2^ with increasing durations from 0.1 to 30 s led to gradual block of beating after each pulse (Figure 3B). The statistical analysis and Hill-fitting of individual experiments showed a sigmoidal dependence of blocking effect on logarithm of pulse duration with a half maximal effect at 1.2 ± 0.4 s (*n* = 6) and maximal effect at ~ 10 s (averaged data in Figure 3D). To compare both gradual stimulation protocols, we calculated and overlaid the total light energy in each light pulse as s^*^μW/mm^2^ (Figure 3E). Surprisingly, we found that the light intensity protocol seemed to be more effective than the pulse duration protocol (as shown in discussion).

To determine if light sensitivity and blocking efficiency is constant, we applied two brief (0.5 s) subthreshold pulses at 100 μW/mm^2^ with only 20 s in-between. Because the blocking effect of the second light pulse was not different (Figures 3F,G, *n* = 5, *p* = 0.46), we conclude that light sensitivity was similar and the submaximal blocking effect was not compromised by LWO refractoriness at this pulse interval.

### LWO Illumination Has Much Higher Temporal Precision Than Agonist Perfusion

**Figure 4:**
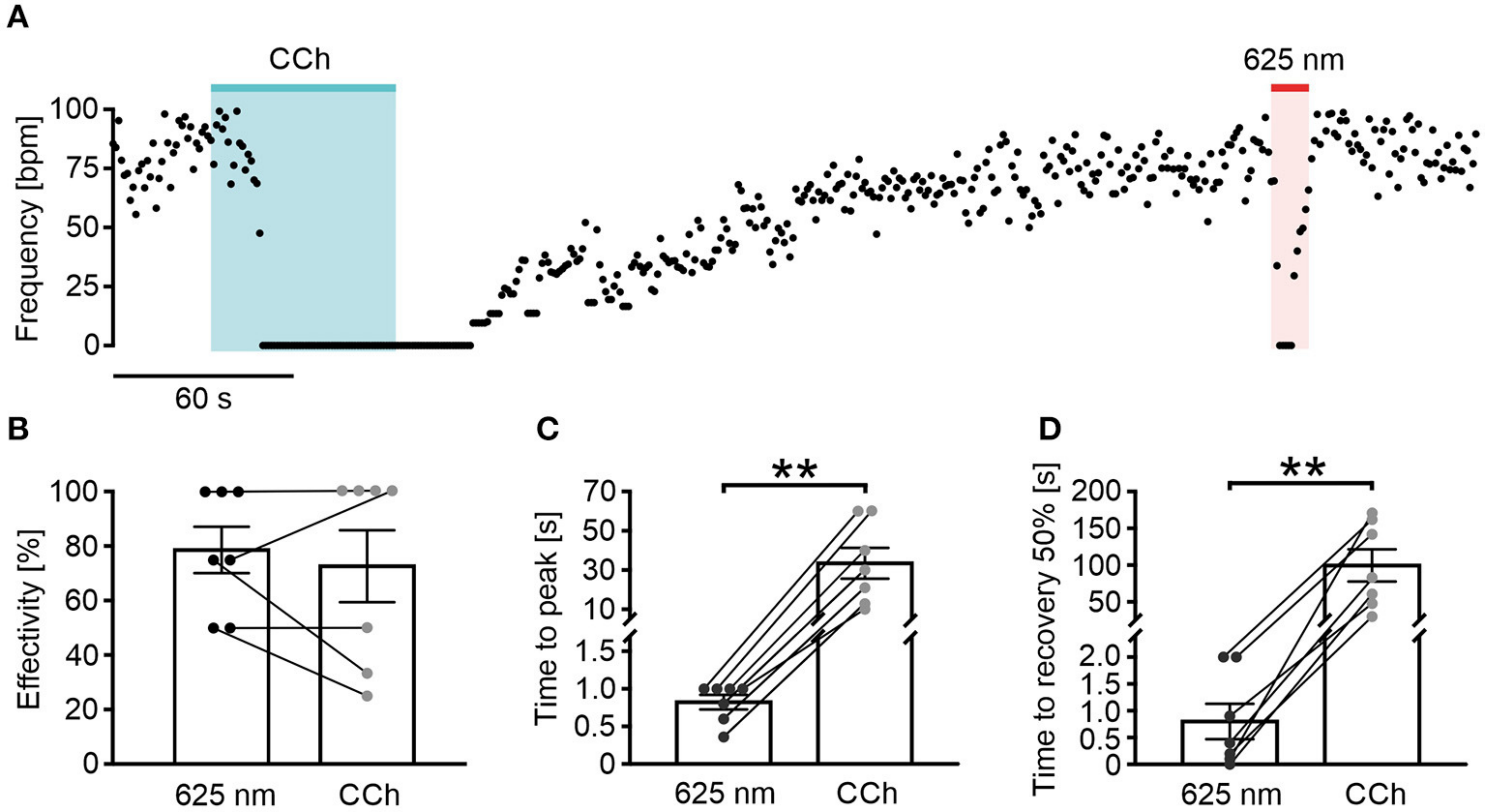
Long wavelength-sensitive cone opsin has higher temporal precision than pharmacological G_i_ stimulation. **(A)** Representative frequency trace of an LWO-EB upon perfusion with carbachol (CCh, 100 μM, 60 s, blue bar) and light stimulation (625 nm, 10 s, 100 μW/mm^2^, red line). **(B–D)** Statistical comparison between CCh and light application for frequency reduction effectivity [**(B)**, paired *t*-test: *p* = 0.49, *n* = 7], time to maximal frequency reduction [**(C)**, paired *t*-test: **p = 0.006, *n* = 7], and time to 50% of beating frequency recovery [**(D)**, paired *t*-test: **p = 0.0039, *n* = 7].

To illustrate the temporal precision of LWO and the advantage over agonist perfusion, we compared the LWO effect with pharmacological stimulation of the G_i_ pathway (Figure 4A) by illumination (LWO, 625 nm) and perfusion of EBs with the acetylcholine-receptor agonist carbachol (CCh, 100 μM). Both stimulations led to similar blocking effectivity (Figure 4B, LWO 78.6 ± 8.5%, *n* = 7, CCh 72.6 ± 13.2%, *n* = 7), however, activation kinetics (delay to maximum block, Figure 4C: LWO 0.8 ± 0.1 s, *n* = 7: CCh 33.4 ± 7.8s, *n* = 7) and deactivation kinetics (time from end of stimulation to 50% recovery, Figure 4D, LWO 0.8 ± 0.3 s, *n* = 7; CCh 99.6 ± 21.9 s, *n* = 7) was significant and up to two orders of magnitude faster using LWO illumination compared with CCh perfusion.

## Discussion

Optogenetic methods have many advantages over pharmacological stimulation, because they allow modulation of G-protein signaling of specific cell types with high temporal and spatial precision using light instead of receptor ligands. The aim of this study was to explore the use of LWO for optogenetic activation of G_i_-signaling in cardiomyocytes. For this purpose, we used stem-cell derived cardiomyocytes which resemble an early embryonic phenotype and show spontaneous beating in cell culture. Importantly, in these cells, the pacemaking mechanism is already well-controlled by G-protein signaling (Boheler et al., 2002; Touhara et al., 2016), such as G_q_, G_s_, and G_i_ proteins (Layden et al., 2010; Beiert et al., 2014; Makowka et al., 2019).

### Rationale for Using LWO to Control G_i_ Signaling

To modulate G_i_ signaling in cardiomyocytes by light, we have chosen the LWO, which has been shown to activate G_i/o_ proteins in HeLa cells (Karunarathne et al., 2013) as well as cultured neurons and the brain of mice (Masseck et al., 2014). Importantly, LWO is well-suited for the repetitive and long-lasting activation of G_i_-dependent GIRK currents by light in HEK293 cells and neurons without desensitization (Masseck et al., 2014). This is of great advantage over vertebrate Rhodopsin, which also activates G_i_ signaling but the responses decline during repetitive stimulation (Masseck et al., 2014). We have chosen LWO over short-wavelength opsin (activated by 350–450 nm) because LWO can be activated by red light >600 nm, which is not absorbed by myoglobin and hemoglobin, penetrates deeper into cardiac tissue (Bruegmann et al., 2016), and has less phototoxic effects. Furthermore, red light >600 nm is spectrally compatible with blue light-activated optogenetic tools, such as Channelrhodopsin-2 for optical depolarization of cardiomyocytes (Bruegmann et al., 2010) or Jellyfish Opsin for light-induced stimulation of the G_s_ signaling cascade (Makowka et al., 2019) which are both not activated by red light and therefore can be combined with LWO co-expression.

### LWO Is G_i_ Specific in Cardiomyocytes and Activates GIRK Channels

It is known that GPCR can show a promiscuous behavior and thereby activate multiple, even counteracting signaling pathways. For instance, Melanopsin, a photoreceptor of intrinsically photosensitive retinal ganglion cells, has been initially described as a G_q_ coupled optogenetic GPCR (Qiu et al., 2005) but it was shown later that it can signal both to G_q_ and G_i_ proteins (Bailes and Lucas, 2013) and can thus activate GIRK channels by G_i_ βγ subunits (Spoida et al., 2016). Our model of spontaneous beating ES-cell derived cardiomyocytes is ideal to discriminate between G_q_ and G_i_ signaling as the former will increase beating rate through PLC/IP_3_/Ca^2+^ release mechanisms enhancing the Ca^2+^ clock pacemaking machinery (Lakatta and DiFrancesco, 2009; Beiert et al., 2014), whereas the latter will reduce beating through G_i_ proteins (Lyashkov et al., 2009). As we have exclusively observed frequency reduction or even complete block of beating by LWO activation (Figure 1D), LWO signals through G_i_ proteins in cardiomyocytes. To confirm this, we blocked G_i_ proteins with PTX that completely abolished all light effects. Notably, we never observed a slightest frequency increase by light in PTX treated LWO EBs (Figure 2B), therefore, excluding G_q_ activation by LWO.

Activated G_i_ proteins can reduce beating rate by two mechanisms: block of adenylate cyclases by G_i_ α subunits with subsequent lowering of PKA-dependent phosphorylation or activation of GIRK potassium channels by G_i_ βγ subunits (Lyashkov et al., 2009). In our experiments, application of the specific GIRK channel inhibitor tertiapin did not affect basal beating rate but reduced the effect of LWO illumination by ~75% suggesting that the major G_i_ effect in ES-cell derived cardiomyocytes obtained by our differentiation protocol is through G_i_ βγ GIRK channel activation. In contrast, GIRK-based reduction of heart rate in mouse sinus nodal cells is only responsible for ~50% of heart rate regulation (Mesirca et al., 2013) presumably reflecting the more robust pacemaking machinery in these cells or differences in GIRK expression. The remaining ~25% of regulation we observed is most likely due to G_i_ α-dependent reduction of basal adenylate cyclase and PKA activity, which has been shown to reduce HCN/I_f_ currents (Abi-Gerges et al., 2000) and L-type-Ca^2+^ currents (Ji et al., 1999) in ES-cell derived cardiomyocytes.

### LWO Overexpression Has No Negative Side-Effects

Overexpression of artificial light sensitive proteins might have negative side effects on the intracellular signaling machinery because of dark activity, G-protein binding, alteration of microdomain signaling, or other overexpression artifacts. In our experience, the spontaneous beating rate is a very sensitive parameter for such side effects. Similar to the expression of Melanopsin for optogenetic G_q_ stimulation (Beiert et al., 2014) and Jellyfish Opsin for optogenetic G_s_ stimulation (Makowka et al., 2019), we did not observe effects on basal beating rate by LWO expression. Since we did not analyze protein expression levels or performed RNA sequencing analysis in this work, we cannot fully exclude minor side effects. However, it must be admitted that the ES-cell system itself cannot exclude side effects for future *in vivo* applications and thus generation of *in vivo* models of LWO overexpression in the heart will be an important next step.

### Fast Kinetics and Light Sensitivity of LWO

Parasympathetic stimulation of the intact heart can be very fast which has been shown for both electrical stimulation of the vagal nerve with ~1.5 s delay (Ng et al., 2001) and optogenetic stimulation of parasympathetic neurons (Moreno et al., 2019) with an immediate reduction of heart rate. In contrast, diffusion-limited pharmacological activation of M2-cholinergic receptors with CCh in the three-dimensional cardiac body *in vitro* resulted in slow activation (~30 s) and deactivation (~100 s). In this *in vitro* setting stimulation of LWO led to 30–100 times faster effects with delays of only 0.8 s from the start of illumination to maximal effect or from end of illumination to 50% of recovery, which is similar to the *in vivo* kinetics. Thereby LWO allows the application of brief continuous light pulses with gradually increasing stimulation intensities; such a stimulation protocol would be almost impossible with slow agonist perfusion and wash out, especially in multicellular preparation, such as EB or in intact organs.

We found that gradual stimulation allows fine tuning the response of G_i_-stimulation on pacemaking activity by either changing pulse duration or light intensity with a sigmoidal dependence on the logarithm of light energy (duration ^*^ intensity in s^*^μW/mm^2^). Interestingly, the half-maximal light energy was slightly higher using variations of intensity (24 s^*^μW/mm^2^) than pulse duration (120 s^*^μW/mm^2^). Specifically, for a fixed total light energy at mid-sensitivity (e.g., 30 s^*^μW/mm^2^, green line in Figure 3E), longer light pulses (10 s at 3 μW/mm^2^, light intensity protocol) are more effective than shorter light pulses (0.3 s at 100 μW/mm^2^, pulse duration protocol) indicating that temporal integration of G_i_ signaling is affecting the threshold blocking effect.

Compared with previous reported LWO sensitivity for GIRK activation in HEK293 cells (4 s^*^μW/mm^2^, Eli50 at 590 nm: 0.2 s, 20 μW/mm^2^, Masseck et al., 2014), we observed lower LWO sensitivity (14–80 s^*^μW/mm^2^ at 625 nm), which could be due to difference in the wavelength used.

The calculation of total light energy allows comparison with other optogenetic GPCR, we have employed to modulate pacemaking of ES-cell derived cardiomyocytes. Using Melanopsin to accelerate pacemaking by G_q_/PLC/IP_3_ signaling, we determined a half maximal energy of 2.4 s^*^μW/mm^2^ (Eli50 of 40 nW/mm^2^ at 60 s pulses, Beiert et al., 2014) and using Jellyfish Opsin to stimulate G_s_/cAMP/PKA signaling, we determined a half maximal energy of only 0.16 s^*^μW/mm^2^ (Eli50 of 8 nW/mm^2^ at 20 s pulses Makowka et al., 2019). Thus, LWO is less light sensitive than Melanopsin and Jellyfish Opsin, which is also advantageous in experimental handling at normal lab room light. In addition, LWO kinetics on frequency reduction (time to peak and 50% deactivation ~0.8 s) was much faster than kinetics of frequency increase by Melanopsin or Jellyfish Opsin in EBs (activation ~10–50 s) which underscores the involvement or fast GIRK channels in G_i_ signaling.

### Outlook

In the future, the combination of LWO with the spectrally compatible JellyOP in transgenic mice (Makowka et al., 2019) will allow spatially confined simultaneous or alternating activation of G_i_ and G_s_ signaling in cardiomyocytes in the heart *in vivo*. This will be a valid approach to study the impact of balanced, dysbalanced, and wrong timing of parasympathetic and sympathetic input which seems to be important for the development of pathologies, such as atrial fibrillation (Ang et al., 2012). The high temporal precision of light enables to study short-term (seconds) and mid-term (minutes and hours) effects and, using implantable light emitting devices, also long-term (days and weeks) chronic G_i_ and G_s_ signaling. Furthermore, selective illumination of left and right ventricle, or of epicardial and endocardial cardiomyocytes will allow to determine regional differences of vegetative nerve input on cardiac function, arrhythmia generation of development of cardiac hypertrophy. Finally, optogenetics has the advantage of cell type-specific expression using specific promoters. Thus, using the Cre-LoxP system, expression of LWO or JellyOpsin in the different cells of the heart (cardiomyocytes, fibroblast, endothelial cells, and smooth muscle cells) will enable investigation of their G_i_- and G_s_-signaling *in vivo* which cannot be performed by agonists applied to the circulation or by electrical stimulation of vegetative nerves.

## Conclusion

Long wavelength-sensitive cone opsin enables optogenetic stimulation of G_i_-signaling cascade in ES-cell derived cardiomyocytes with red light, resulting in a high effective and very fast inhibition of spontaneous pacemaking, mainly through the activation of GIRK channels. Thus, LWO itself or in combination with the G_s_ coupled spectrally compatible optogenetic GPCR JellyOP will allow to investigate the physiological and pathological effects of balanced and dysbalanced vegetative nerve input on the heart.

## Data Availability Statement

The raw data supporting the conclusions of this article will be made available by the authors, without undue reservation.

## Author Contributions

MC, DM, TB, and PS designed the research. MC performed the research. MC, DM, and PS wrote the manuscript. All authors contributed to the article and approved the submitted version.

## Funding

This work was supported by the Deutsche Forschungsgemeinschaft (DFG, German Research Foundation)—313904155/SA 1785/7-1, 380524518/SA1785/9-1, and 214362475/GRK1873/2.

## Conflict of Interest

The authors declare that the research was conducted in the absence of any commercial or financial relationships that could be construed as a potential conflict of interest.

## Publisher's Note

All claims expressed in this article are solely those of the authors and do not necessarily represent those of their affiliated organizations, or those of the publisher, the editors and the reviewers. Any product that may be evaluated in this article, or claim that may be made by its manufacturer, is not guaranteed or endorsed by the publisher.
